# Supplementary material for: Mapping Robusta coffee (Coffea canephora) cropping systems in Uganda: A two-step pixel and sub-pixel based approach with Sentinel-2 data
Source: PLoS One. 2026 Jan 5;21(1):e0338803. doi: 10.1371/journal.pone.0338803 (PMC12768349; doi:10.1371/journal.pone.0338803)
Supplement: Attachment — Submitted filename: Review_report-PONE-D-25-11912_reviewer.pdf [file pone.0338803.s001.pdf]

Consider revising the title to indicate the two steps involved. Also, the rationale for mapping Robusta coffee CS in Uganda is not articulated in the abstract. The abstract's last sentence should state how the findings could be applied.

The citation on line 46 [Okia & Buyinza (2022)] is not a numbered reference.

In line 63, 'African' should be 'Africa.' For lines 65–66, consider: 'For instance, the spatial resolution of Landsat (30 m) and Sentinel-2 (10 m) imagery…'

In line 67, delete 'in Africa'; coffee is grown on a small scale in other parts of the world. Also, replace 'hidden' with 'subtle'

Lines 83–87: Revise the objective to: 'This study evaluates the performance of MESMA in mapping three distinct Robusta coffee systems using Sentinel-2 imagery in Uganda.'

Lines 95–96: Remember that, for *29° 31' W* Uganda is in the Eastern Hemisphere. Also, on line 95, the minute symbol is incorrectly placed on 32. All maps should use a consistent coordinate grid format; the one in the Results is different from that described in the Methods section.

Ensure that all maps in the manuscript include coordinate grids for spatial reference. The caption for Figure 1 is long; consider moving some of the descriptive information to the main text. Is Uganda an island surrounded by water?

Significant class imbalance is evident; how did the study handle this challenge to balance training data? Also, the 'mixed agricultural and grassland' class is too broad and heterogeneous, possibly increasing spectral confusion.

After describing the data used in the study, it is advisable to include a table summarizing the datasets along with their properties.

The RF classifier uses default parameters, which may not yield optimal performance without tuning. Also, there is no detail on how training and validation samples were selected.

K-Means may not be the most reliable method for endmember extraction, particularly in mixed pixels. The method used assigns pixels to the class with the highest fraction, this can be problematic when classes are spectrally similar or mixed. Report fractional abundance maps and threshold sensitivity to show robustness. You may consider fuzzy accuracy assessments especially when working with sub-pixel classification.

Sentinel-2's spatial resolution may not adequately resolve smallholder Robusta plots or intercropping patterns. Higher-resolution data such as PlanetScope may address potential limitations in mapping under canopy or mixed vegetation.

The statement 'Natural forests, mixed agricultural and grassland cover 75.1% of the total area' is misleading, as the table indicates that natural forest covers 708,886.7 ha (7.5%) and mixed agricultural and grassland covers 7,118,342.6 ha (75.1%).

There is no critical discussion of cases where certain classes have lower UA, or PA compared to others. Also, although the MESMA average RMSE is low, areas with higher RMSE should be analyzed.

To confirm robust separability for all pixels, it would strengthen the analysis to include a measure such as the spectral angle mapper (SAM) or a spectral separability index.

The thresholds used to consider a pixel 'dominated' by a given cropping system should be stated and justified.

There is no discussion on which vegetation indices were most important or how they improved classification performance.

The manuscript alternates between 'Robusta coffee CS,' 'Robusta CS,' and 'Robusta coffee.'

The conclusion should not just reiterate points already made without adding new insights. Provide a short concluding paragraph that sets the stage for future research.

In some sections, such as lines 114–119, the manuscript contains long sentences that may be difficult to understand. Consider splitting these into shorter, clearer sentences.

The manuscript requires significant language editing!
