# Supplementary material for: Mapping Robusta coffee (Coffea canephora) cropping systems in Uganda: A two-step pixel and sub-pixel based approach with Sentinel-2 data
Source: PLoS One. 2026 Jan 5;21(1):e0338803. doi: 10.1371/journal.pone.0338803 (PMC12768349; doi:10.1371/journal.pone.0338803)
Supplement: Attachment — Submitted filename: Response to Reviewers.docx [file pone.0338803.s002.docx]

Date: July 23, 2025

Dr. Emily Chenette,

Editor-in-Chief

Journal of PLOS One California, USA

Mr. Getachew Kebede

Data Management, Modelling, and Geoinformation (DMMG) Unit

Tel.: +254208632280

Mob.: +254790224476

Email: [garagaw@icipe.org](mailto:garagaw@icipe.org)

**Subject: Submission of revised paper (Manuscript Number: PONE-D-25-11912)**

Dear Dr. Emily Chenette,

We appreciate your time in handling our above-referenced manuscript entitled “Mapping Robusta coffee (*Coffea canephora*) cropping systems in Uganda: A two-step approach with Sentinel-2. We are also grateful for the valuable feedback provided by the editor and the reviewers.

We have carefully revised the manuscript and addressed all comments from the editor and reviewers. In the revised version, changes are highlighted in turquoise (Editor), green (Reviewer #1), and yellow (Reviewer #2). We hope that our revision and responses are adequate and sufficient.

Sincerely,

Getachew Kebede Aragaw

Corresponding author

**Editorial Request – Detailed point-by-point response**

**Editorial Request #1:**

When submitting your revision, we need you to address these additional requirements.

1. Please ensure that your manuscript meets PLOS ONE's style requirements, including those for file naming. The PLOS ONE style templates can be found at https://journals.plos.org/plosone/s/file?id=wjVg/PLOSOne_formatting_sample_main_body.pdf and <https://journals.plos.org/plosone/s/file?id=ba62/PLOSOne_formatting_sample_title_authors_affiliations.pdf>

**Authors’ response**

We have revised the manuscript to comply with PLOS ONE’s formatting and file naming requirements, following the provided templates. All files have been updated accordingly.

**Editorial Request #2:**

Please include a complete copy of PLOS’ questionnaire on inclusivity in global research in your revised manuscript. Our policy for research in this area aims to improve transparency in the reporting of research performed outside of researchers’ own country or community. The policy applies to researchers who have travelled to a different country to conduct research, research with Indigenous populations or their lands, and research on cultural artefacts. The questionnaire can also be requested at the journal’s discretion for any other submissions, even if these conditions are not met.  Please find more information on the policy and a link to download a blank copy of the questionnaire here: <https://journals.plos.org/plosone/s/best-practices-in-research-reporting>.

Please upload a completed version of your questionnaire as Supporting Information when you resubmit your manuscript.

**Authors’ response**

We have completed the PLOS inclusivity in global research questionnaire and included it as Supporting Information with our revised submission, as requested.

**Editorial Request #3:**

In your Methods section, please provide additional information regarding the permits you obtained for the work. Please ensure you have included the full name of the authority that approved the field site access and, if no permits were required, a brief statement explaining why.

**Authors’ response**

We have updated the Methods section to include the full names of the authorities. Field data collection was authorized by the Uganda National Coffee Research Institute (NaCORI) and the National Forestry Resources Research Institute (NaFORRI), the national agencies responsible for overseeing coffee and forest research in Uganda.

**Editorial Request #4:**

Please note that PLOS ONE has specific guidelines on code sharing for submissions in which author-generated code underpins the findings in the manuscript. In these cases, we expect all author-generated code to be made available without restrictions upon publication of the work. Please review our guidelines at <https://journals.plos.org/plosone/s/materials-and-software-sharing#loc-sharing-code>

and ensure that your code is shared in a way that follows best practice and facilitates reproducibility and reuse.

**Authors’ response**

We have reviewed the PLOS ONE code sharing guidelines and made all author-generated code publicly available in accordance with best practices. The code has been uploaded as Supporting Information and is also accessible via the *icipe* Data Portal at the following link:

<https://dmmg.icipe.org/dataportal/dataset/mapping-robusta-coffee-coffea-canephora-cropping-systems-in-uganda>.

This ensures transparency, reproducibility, and unrestricted access upon publication.

**Editorial Request #5:**

Thank you for stating in your Funding Statement:

 [Getachew Kebede was supported by the In-Region Postgraduate Scholarship from the German Academic Exchange Service (DAAD). The authors sincerely appreciate the financial support provided by the European Union (EU) for the project “Robusta coffee agroforestry to adapt and mitigate climate change in Uganda” (GCCA+-Global Climate Change Alliance & DESIRA, Project/Grant No. FOOD/2021/427-759). Additional funding was received from the Swedish International Development Cooperation Agency (Sida), the Swiss Agency for Development and Cooperation (SDC), the Australian Centre for International Agricultural Research (ACIAR), the Norwegian Agency for Development Cooperation (Norad), the Federal Democratic Republic of Ethiopia, and the Government of the Republic of Kenya. The project also benefited from funding by the Centre de Coopération Internationale en Recherche Agronomique pour le Développement (CIRAD).].

Please provide an amended statement that declares *all* the funding or sources of support (whether external or internal to your organization) received during this study, as detailed online in our guide for authors at http://journals.plos.org/plosone/s/submit-now.

Please also include the statement “There was no additional external funding received for this study.” in your updated Funding Statement.

Please include your amended Funding Statement within your cover letter. We will change the online submission form on your behalf.

**Authors’ response**

We have amended the Funding Statement to declare all sources of support, both external and internal, and included the required sentence: “There was no additional external funding received for this study.”

Amended Funding Statement:

Getachew Kebede was supported by the In-Region Postgraduate Scholarship from the German Academic Exchange Service (DAAD). The authors gratefully acknowledge financial support from the European Union (EU) for the project “Robusta coffee agroforestry to adapt and mitigate climate change in Uganda” (GCCA+ - Global Climate Change Alliance & DESIRA, Project/Grant No. FOOD/2021/427-759). Additional funding was received from the Swedish International Development Cooperation Agency (Sida), the Swiss Agency for Development and Cooperation (SDC), the Australian Centre for International Agricultural Research (ACIAR), the Norwegian Agency for Development Cooperation (Norad), the Federal Democratic Republic of Ethiopia, the Government of the Republic of Kenya, and the Centre de Coopération Internationale en Recherche Agronomique pour le Développement (CIRAD). Internal support was also provided by the authors’ host institutions through logistical, technical, and administrative assistance. There was no additional external funding received for this study.

**Editorial Request #6:**

Thank you for uploading your study's underlying data set. Unfortunately, the repository you have noted in your Data Availability statement does not qualify as an acceptable data repository according to PLOS's standards.

At this time, please upload the minimal data set necessary to replicate your study's findings to a stable, public repository (such as figshare or Dryad) and provide us with the relevant URLs, DOIs, or accession numbers that may be used to access these data. For a list of recommended repositories and additional information on PLOS standards for data deposition, please see [https://journals.plos.org/plosone/s/recommended-repositories](https://eur02.safelinks.protection.outlook.com/?url=https%3A%2F%2Ftrack.editorialmanager.com%2FCL0%2Fhttps%3A%252F%252Fjournals.plos.org%252Fplosone%252Fs%252Frecommended-repositories%2F1%2F010f01971d8e9b71-10309e21-6007-494a-a279-e37627068eed-000000%2FE7MOEOB_lf4vAevkFfdqwkN-V3mWtYCEZVHWrvXrex0%3D213&data=05%7C02%7Cgaragaw%40icipe.org%7Cd986aa607a2c4ef3ac8d08dd9ee88b50%7Ca4e98fb557d94b97b7b260ab87e9a234%7C0%7C0%7C638841444231677167%7CUnknown%7CTWFpbGZsb3d8eyJFbXB0eU1hcGkiOnRydWUsIlYiOiIwLjAuMDAwMCIsIlAiOiJXaW4zMiIsIkFOIjoiTWFpbCIsIldUIjoyfQ%3D%3D%7C0%7C%7C%7C&sdata=L4JFXX8YJU1%2Bk72EEWVxvBnwbxdUbyle0P2X4fOy57U%3D&reserved=0).

When completing the data availability statement of the submission form, you indicated that you will make your data available on acceptance. We strongly recommend all authors decide on a data sharing plan before acceptance, as the process can be lengthy and hold up publication timelines. Please note that, though access restrictions are acceptable now, your entire data will need to be made freely accessible if your manuscript is accepted for publication. This policy applies to all data except where public deposition would breach compliance with the protocol approved by your research ethics board. If you are unable to adhere to our open data policy, please kindly revise your statement to explain your reasoning and we will seek the editor's input on an exemption. Please be assured that, once you have provided your new statement, the assessment of your exemption will not hold up the peer review process.

We note that Figures 1, 3, 6 and 7 in your submission contain [map/satellite] images which may be copyrighted. All PLOS content is published under the Creative Commons Attribution License (CC BY 4.0), which means that the manuscript, images, and Supporting Information files will be freely available online, and any third party is permitted to access, download, copy, distribute, and use these materials in any way, even commercially, with proper attribution. For these reasons, we cannot publish previously copyrighted maps or satellite images created using proprietary data, such as Google software (Google Maps, Street View, and Earth). For more information, see our copyright guidelines: http://journals.plos.org/plosone/s/licenses-and-copyright.

We require you to either (1) present written permission from the copyright holder to publish these figures specifically under the CC BY 4.0 license, or (2) remove the figures from your submission:

1. You may seek permission from the original copyright holder of Figures 1, 3, 6 and 7 to publish the content specifically under the CC BY 4.0 license.

We recommend that you contact the original copyright holder with the Content Permission Form (http://journals.plos.org/plosone/s/file?id=7c09/content-permission-form.pdf) and the following text:

“I request permission for the open-access journal PLOS ONE to publish XXX under the Creative Commons Attribution License (CCAL) CC BY 4.0 (http://creativecommons.org/licenses/by/4.0/). Please be aware that this license allows unrestricted use and distribution, even commercially, by third parties. Please reply and provide explicit written permission to publish XXX under a CC BY license and complete the attached form.”

Please upload the completed Content Permission Form or other proof of granted permissions as an "Other" file with your submission.

In the figure caption of the copyrighted figure, please include the following text: “Reprinted from [ref] under a CC BY license, with permission from [name of publisher], original copyright [original copyright year].”

If you are unable to obtain permission from the original copyright holder to publish these figures under the CC BY 4.0 license or if the copyright holder’s requirements are incompatible with the CC BY 4.0 license, please either i) remove the figure or ii) supply a replacement figure that complies with the CC BY 4.0 license. Please check copyright information on all replacement figures and update the figure caption with source information. If applicable, please specify in the figure caption text when a figure is similar but not identical to the original image and is therefore for illustrative purposes only.

The following resources for replacing copyrighted map figures may be helpful:

USGS National Map Viewer (public domain): http://viewer.nationalmap.gov/viewer/

The Gateway to Astronaut Photography of Earth (public domain): http://eol.jsc.nasa.gov/sseop/clickmap/

Maps at the CIA (public domain): https://www.cia.gov/library/publications/the-world-factbook/index.html and https://www.cia.gov/library/publications/cia-maps-publications/index.html

NASA Earth Observatory (public domain): http://earthobservatory.nasa.gov/

Landsat: http://landsat.visibleearth.nasa.gov/

USGS EROS (Earth Resources Observatory and Science (EROS) Center) (public domain): http://eros.usgs.gov/#

Natural Earth (public domain): [http://www.naturalearthdata.com/](https://eur02.safelinks.protection.outlook.com/?url=http%3A%2F%2Ftrack.editorialmanager.com%2FCL0%2Fhttp%3A%252F%252Fwww.naturalearthdata.com%252F%2F1%2F010f01971d8e9b71-10309e21-6007-494a-a279-e37627068eed-000000%2FzDHUrFp8onlq-dBg7WFzcN94TE42Ucytv-EnwRm0AyQ%3D213&data=05%7C02%7Cgaragaw%40icipe.org%7Cd986aa607a2c4ef3ac8d08dd9ee88b50%7Ca4e98fb557d94b97b7b260ab87e9a234%7C0%7C0%7C638841444231698895%7CUnknown%7CTWFpbGZsb3d8eyJFbXB0eU1hcGkiOnRydWUsIlYiOiIwLjAuMDAwMCIsIlAiOiJXaW4zMiIsIkFOIjoiTWFpbCIsIldUIjoyfQ%3D%3D%7C0%7C%7C%7C&sdata=2uQBfkco9u%2B506r%2FzSf%2FfOVIXSa2cQ2Sr1Hvu9xscPE%3D&reserved=0)

**Authors’ response**

We have reviewed the PLOS ONE code sharing guidelines and made all author-generated code publicly available in accordance with best practices. The code has been uploaded as Supporting Information and is also accessible via the *icipe* Data Portal at the following link:

<https://dmmg.icipe.org/dataportal/dataset/mapping-robusta-coffee-coffea-canephora-cropping-systems-in-uganda>.

This ensures transparency, reproducibility, and unrestricted access upon publication.

**A**ll data in Figure 1 were obtained from open sources that are published in the public domain: Natural Earth (public domain): <http://www.naturalearthdata.com/> and USGS EROS (Earth Resources Observatory and Science (EROS) Center) (public domain): [http://eros.usgs.gov/#](http://eros.usgs.gov/).

For Figures 3, 6, and 7, the shape files were sourced from Natural Earth (public domain): <http://www.naturalearthdata.com/>, whereas the satellite data in these figures were obtained from Sentunel 2: [https://dataspace.copernicus.eu/explore-data/data-collections/sentinel-data/sentinel-2](https://eur02.safelinks.protection.outlook.com/?url=https%3A%2F%2Fdataspace.copernicus.eu%2Fexplore-data%2Fdata-collections%2Fsentinel-data%2Fsentinel-2&data=05%7C02%7Ceabdel-Rahman%40icipe.org%7Cc1bada6007734d31727608ddbefbbaf4%7Ca4e98fb557d94b97b7b260ab87e9a234%7C0%7C0%7C638876710556775742%7CUnknown%7CTWFpbGZsb3d8eyJFbXB0eU1hcGkiOnRydWUsIlYiOiIwLjAuMDAwMCIsIlAiOiJXaW4zMiIsIkFOIjoiTWFpbCIsIldUIjoyfQ%3D%3D%7C0%7C%7C%7C&sdata=E7Mfr3QfXFl1N7PlJXB9IcZlmp%2Fo8KiOq3Bdiy3J2LQ%3D&reserved=0) through Google Earth Engine <https://doi.org/10.1016/j.rse.2017.06.031>.

The Sentinel 2 data are publicly and legally available to anyone worldwide, with no restrictions on commercial or non-commercial use under the open-access compliant Creative Commons Attribution-ShareAlike 3.0 IGO (CC BY-SA 3.0 IGO) license ([https://open.esa.int/copernicus-sentinel-satellite-imagery-under-open-licence](https://eur02.safelinks.protection.outlook.com/?url=https%3A%2F%2Fopen.esa.int%2Fcopernicus-sentinel-satellite-imagery-under-open-licence&data=05%7C02%7Ceabdel-Rahman%40icipe.org%7C6ffed367e4da4ff7061508ddc043916b%7Ca4e98fb557d94b97b7b260ab87e9a234%7C0%7C0%7C638878118611252792%7CUnknown%7CTWFpbGZsb3d8eyJFbXB0eU1hcGkiOnRydWUsIlYiOiIwLjAuMDAwMCIsIlAiOiJXaW4zMiIsIkFOIjoiTWFpbCIsIldUIjoyfQ%3D%3D%7C0%7C%7C%7C&sdata=fdCJAKYks09iRbGI0fbRuBoTWrnvwWmpbSFCjg8C%2Fcs%3D&reserved=0)).

**Editorial Request #7:**

Please remove your figures from within your manuscript file, leaving only the individual TIFF/EPS image files, uploaded separately. These will be automatically included in the reviewers’ PDF.

**Authors’ response**

All figures have been removed from the manuscript file and uploaded separately as individual TIFF/EPS files, as requested.

**Reviewer 1 – Detailed point-by-point response**

**Reviewer 1**

**Comment 1:**

“Consider revising the title to indicate the two steps involved.”

**Authors’ response**

Thank you for the suggestion. We have revised the title to reflect the two-step pixel- and sub-pixel-based approach used in the study. The title no reads “*Mapping Robusta coffee (Coffea canephora) cropping systems in Uganda: A two-step pixel and sub-pixel based approach with Sentinel-2 data*

”

**Comment 2:**

“The rationale for mapping Robusta coffee CS in Uganda is not articulated in the abstract.”

**Authors’ response**

We have revised the abstract to articlaute the rationale for mapping Robusta coffee cropping systems in Uganda (see text highlighted in green).

**Comment 3:**

“The abstract's last sentence should state how the findings could be applied.”

**Authors’ response**

The final sentence of the abstract reads “The findings of this study can inform site-specific interventions (e.g., pest management, fertilizer application, etc.) based on the type of CS, and understanding of how different systems impact coffee productivity” in the revised manauscript.

**Comment 4:**

“The citation on line 46 [Okia & Buyinza (2022)] is not a numbered reference.”

**Authors’ response**

Thank you. We have corrected the citation format in line 61 to align with the journal’s reference style.

**Comment 5:**

“In line 63, 'African' should be 'Africa.’”

**Authors’ response**

Thank you. The correction has been made as suggested (line 87)

**Comment 6:**

“For lines 65–66, consider: ‘For instance, the spatial resolution of Landsat (30 m) and Sentinel-2 (10 m) imagery…’”

**Authors’ response**

Thank you. We have revised the sentence in line 94 for improved clarity and accuracy as suggested by the reviewer.

**Comment 7:**

“In line 67, delete 'in Africa'; coffee is grown on a small scale in other parts of the world. Also, replace 'hidden' with 'subtle.'”

**Authors’ response**

We thank the reviewer for the helpful suggestion. As recommended, we have deleted “in Africa” and replaced “hidden” with “subtle” in lines 91–92.

**Comment 8:**

“Lines 83–87: Revise the objective to: ‘This study evaluates the performance of MESMA in mapping three distinct Robusta coffee systems using Sentinel-2 imagery in Uganda.’”

**Authors’ response**

Thank you for the valuable suggestion. We have revised the objective statement as suggested (lines 109–110).

**Comment 9:**

“Lines 95–96: Remember that, for 29° 31' W Uganda is in the Eastern Hemisphere. Also, on line 95, the minute symbol is incorrectly placed on 32.”

**Authors’ response**

Thank you for the observation. We have corrected the longitude to 29° 31′ E and adjusted the placement of the minute symbol in lines 126–127.

**Comment 10:**

“All maps should use a consistent coordinate grid format; the one in the Results is different from that described in the Methods section.”

**Authors’ response**

Thank you for the comment. We have ensured consistency by processing and displaying all maps using the WGS84 coordinate system in decimal degrees, as now clearly stated in the Methods section (lines 227–229).

**Comment 11:**

“The caption for Figure 1 is long; consider moving some of the descriptive information to the main text. Is Uganda an island surrounded by water?”

**Authors’ response**

We sincerely thank the reviewer for the helpful comment. The caption for Fig. 1 has been shortened as suggested. Additionally, we have adjusted the background color of neighboring countries to avoid confusion, and we confirm that Uganda is not depicted as an island.

**Comment 12:**

“Significant class imbalance is evident; how did the study handle this challenge to balance training data?”

**Authors’ response**

Thank you for the comment. We have added a few sentences to the Discussion (lines 618–622) highlighting class imbalance as a limitation, especially for underrepresented classes like water bodies and built-up areas, and suggest addressing this in future studies.

**Comment 13:**

“The ‘mixed agricultural and grassland' class is too broad and heterogeneous, possibly increasing spectral confusion.”

**Authors’ response**

Thank you for the comment. We acknowledge that the ‘mixed agricultural and grassland’ class includes diverse land covers, contributing to spectral heterogeneity and overlap. Based on field observations, a clear separation was not feasible in some sites in our study area. This has been highlighted as a shortcoming in the Discussion chapter (lines 548– 55).

**Comment 14:**

“After describing the data used in the study, it is advisable to include a table summarizing the datasets along with their properties.”

**Authors’ response**

Thank you for the suggestion. We have included more information (data source and dates) about dataset in Table 1.

**Comment 15:**

“The RF classifier uses default parameters, which may not yield optimal performance without tuning. Also, there is no detail on how training and validation samples were selected.”

**Authors’ response**

Thank you for the helpful comment. Yes, we agree with the reviewer that when the RF parameters (ntree and mtry) are optimized, the performance of the model may increase. Some other studies such as Breiman, (31) and Liaw & Wiener, (44) found that the performance of RF classifier based on the default ntree and mtry, is not significantly different when these two parameters are tuned and optimized. For the validation details, highlighted it under field data collection in lines 153-154. We thank the reviewer particularly for this comment, which allowed us to thoroughly revise our field data collection.

**Comment 16:**

“K-Means may not be the most reliable method for endmember extraction... Report fractional abundance maps and threshold sensitivity.”

**Authors’ response**

Thank you for the meticulous comment. Yes, you are right, K-means was not used for endmember extraction; it was an oversight in the text. We used fractional abundance to extract endmembers. In the revised manuscript, we included the threshold sensitivity as described in the methodology (lines 363-366), results (lines 458-462). We also provided a discussion argument to interpret these results.

**Comment 17:**

“Sentinel-2’s spatial resolution may not adequately resolve smallholder Robusta plots…”

**Authors’ response**

Thank you for the observation. We included this a a limitation in the revised Discussion (lines 626–633) chapter.

**Comment 18:**

“The statement 'Natural forests, mixed agricultural and grassland cover 75.1%' is misleading…”

**Authors’ response**

Thank you for the observation. We have rephrased the sentence in line 413 – 414

**Comment 19:**

“There is no critical discussion of cases where certain classes have lower UA or PA…”

**Authors’ response**

Thank you for the comment. This point has been addressed in the revised Discussion section (lines 513– 518).

**Comment 20:**

“To confirm robust separability, include spectral angle mapper (SAM) or spectral separability index.”

**Authors’ response**

Thank you for the suggestion. We agreed with the reviewer and included SAM and spectral separability in our revised manuscript. Please see lines 361 –373 in the methods, 462– 467 in the results, and 592–600 in the discussion chapter. .

**Comment 21:**

“Thresholds used to consider a pixel ‘dominated’ by a cropping system should be stated and justified.”

**Authors’ response**

Thank you for the comment. We have added the threshold criteria in the Methods section and justified the selected values based on the distribution of fractional abundance histograms. Relevant revisions were made in the methodology (lines 363-366), results (lines 458-462) and Discussion (lines 585–592).

**Comment 22:**

“No discussion on which vegetation indices were most important or how they improved classification performance.”

**Authors’ response**

Thank you for the comment. We have included this in the discussion (Lines 506–510).

**Comment 23:**

“The manuscript alternates between 'Robusta coffee CS,' 'Robusta CS,' and 'Robusta coffee.'”

**Authors’ response**

Terminology has been standardized to "Robusta coffee CS throughout the manuscript.

**Comment 24:**

“The conclusion should not just reiterate points already made… Provide a short concluding paragraph that sets the stage for future research.”

**Authors’ response**

Thank you for the suggestion. We have revised the conclusion to reduce repetition and included a forward-looking statements (lines 651–658).

**Comment 25:**

“Lines 114–119 contain long sentences; consider breaking them into shorter ones.”

**Authors’ response**

Since we revised the field data collection section, these sentences are also been revised .

**Comment 26:**

“The manuscript requires significant language editing!”

**Authors’ response**

The entire manuscript has undergone thorough language editing to improve clarity, grammar, and coherence.

**Reviewer 2 – Detailed point-by-point response**

**Reviewer 2 – Comment 1:**

Overall Comments: The authors present an interesting and valuable contribution by developing a robusta coffee map for the main growing regions of Uganda. The study employs a two-step approach that combines Random Forest classification with endmember spectral mixture analysis (MESMA), utilizing spectral information collected in the field.
To enhance reproducibility and facilitate comparison with future studies, I recommend that the authors provide a more detailed description of their methodology and improve the validation protocol of their map. Taking into account mapped areas and giving confidence intervals for their figures.

**Authors’ response**

We thank the reviewer for the positive feedback. In response, we have added more detail to the methodology and improved the map validation protocol by incorporating mapped area statistics and confidence intervals to enhance reproducibility and comparison of our results.

**Comment 2:**

Additionally, it would be beneficial if the authors elaborate on the potential applications of this map.

**Authors’ response**

Thank you. In the revised Abstract (lines 22–25) and conclusions we elaborated on the potential application of the coffee maps produced in thsis study.

**Comment 3:**

Finally, in accordance with the journal’s data availability policy, the authors should ensure that all relevant data, namely, the map, calibration/training data, and validation data are made publicly available.

**Authors’ response**

We have reviewed the PLOS ONE code sharing guidelines and made all author-generated code publicly available in accordance with best practices. The code has been uploaded as Supporting Information and is also accessible via the icipe Data Portal at the following link:

<https://dmmg.icipe.org/dataportal/dataset/mapping-robusta-coffee-coffea-canephora-cropping-systems-in-uganda>.

This ensures transparency, reproducibility, and unrestricted access upon publication.

**Comment 4:**

Recommendation:

As per the journal’s policy:

“Authors are required to make all data underlying the findings described fully available, without restriction, and from the time of publication.” Currently, the training and validation datasets, as well as the final map, do not appear to be accessible. Additionally, the spectral data shared online are not self-explanatory or documented making their use difficult.

**Authors’ response**

Once again, we thank the reviewer for this. Please refer to our above responses. Our data are available at

<https://dmmg.icipe.org/dataportal/dataset/mapping-robusta-coffee-coffea-canephora-cropping-systems-in-uganda>.

**Comment 5:**
Section: Abstract:

Line 27: The accuracy figure cited as 87% does not correspond to any value in the main text. The authors likely meant 89.9%.

**Authors’ response**

Your are right, thank you. This was an oversight, and corrected the figure to read 89.9

**Comment 6:**

Section: Introduction:

The introduction presents the situation of coffee in Uganda and the mapping methodologies, but do not make clear connection between the two. Additionally, it would be good to expand on how the map can be used.

**Authors’ response**

Thank you for the comment. We have added a paragraph to reflect and address this valuable comment.

**Comment 7:**

Line 45: The phrase "lagging behind" is vague. Consider specifying which aspects (e.g., productivity, technology adoption, mapping efforts).

**Authors’ response**

Thank you for the helpful comment. It is addressed in line 57 – 60.

**Comment 8**

Line 65: The resolution seems inverted between Sentinel and Landsat

**Authors’ response**

Thank you for pointing this out. We agreed and addressed this (line 94).

**Comment 9:**

Section: Data Collection

This section should be clarified. The sampling protocol is not clear. What is the difference between the ROI and representative pixel? The clustering methodologies should be clearly explained and reproducible. Which climate indicators were used? Which source of data was used for the slope, elevation, rainfall, ... The clustering result should be presented on a map.

**Authors’ response**

Thank you for the comment. We have addressed all these points in our revised manuscript, where we included a table to show the data sources as commented by Reviewer#1, and revised study map.

**Comment 10:**
What is the google earth platform (Goole earth engine, Google earth)? What is the imagery source with a resolution of 30m. Do the authors mean 30cm?

**Authors’ response**

Thank you for your observation. We used Google Earth for digitizing training polygons (ROIs) for major LULC classes, using a Landsat 8 background image (30 m resolution) captured on January 15, 2022, not 30 cm. This has been clarified in the revised manuscript (lines 168 to 172, Data Collection section) to avoid confusion between Google Earth and Google Earth Engine (GEE).

**Comment 11:**
The number of samples used for validation appears imbalanced and insufficient for some classes. For example, only ~20 pixels are used for water bodies and ~16 for built-up areas. These small sample sizes are not statistically robust for accuracy assessment.

**Authors’ response**

Thank you for pointing this out. We used 54 built-up and 68 water body pixels, split 70:30 for training and validation, yielding ~16 and ~20 validation pixels, respectively. Due to their small spatial extent, sample sizes were limited but carefully selected using field data. This limitation is now acknowledged in the manuscript (Discussion, lines 618–622), with a recommendation to increase samples for minor classes in future studies, as also noted by Reviewer #1.

**Comment 12:**
Further, more information is needed on the size of the digitalized polygons. There may be spatial correlation between training and validation samples if they are geographically close, which could artificially inflate accuracy metrics. A figure showing digitalized area could help the reader understand the method.

**Authors’ response**

Thank you for the comment. We have now included a map showing the distribution of the polygons. Please see lines 202 to 208 and Fig. 2, which address this shortfall. We thank the reviewer once again for this particular suggestion.

**Comment 13:**

Section: Methods

The categories choice should be discussed. It should be clarified in which categories other tree crops which could be confused with coffee are grouped. The possible presence of arabica coffee should also be addressed.

**Authors’ response**

Thank you for the comment. The cropping system categories are based on our previous work (Kebede et al., 2024), where the classification criteria are detailed. This has been clarified in the Methods section (lines 301–311) and further addressed in the Discussion (lines 622–625) in response to this specific comment.

**Comment 14:**

Line 142: Is the composite made of 2 images or all the January and February images?

**Authors’ response**

We thank the reviewer for the observation. The composite was generated using all available Sentinel-2 images from January 1 to February 28, 2023, not just two specific dates edited in line 242

**Comment 15:**

Line 157: Pre-processing steps need to be described in more detail to ensure reproducibility (e.g., cloud masking, atmospheric correction, resampling procedures).

**Authors’ response**

Thank you for the suggestion. We have added further details on the pre-processing steps, particularly the resampling procedures, to enhance reproducibility. These revisions are included in the updated Methods section (lines 245 to 253).

**Comment 16:**

Section: Endmember Selection and Spectral Data Collection

A brief description of the spectral data collection process should be provided. The text should clearly state that data were collected during the dry season.

**Authors’ response**

Thank you. We have added a brief description of the spectral data collection and procedures are now detailed in lines 313 to 327 (2.2.2 Endmembers selection and spectral data collection section).

**Comment 17:**

The author should address how/why those systems were selected. Some comment on the fact that the endmembers are not “pure” as they will all have some of the spectra of robusta plantation. The meaning of decomposing a pixel in Robusta full sun and "banana + robusta” should be explained as robusta appear in the two classes. Naively I would expect that each pixel is decomposed into robusta, banana and other types of agroforestry so there is a clear sum to 1. i.e 60% robustra+40% banana. Finally, the “robusta+agroforestry” category seems ill defined had it is a mix of different systems with different spectra. The authors should specify which agroforestry systems are included in this category.

**Authors’ response**

Thank you for the comment. The cropping system categories are based on our previous work (Kebede et al., 2024), where the classification criteria are detailed. This has been clarified in the Methods (lines 301–311).

**Comment 18:**

Section: Robusta coffee cropping systems (CS) discrimination using multiple endmember spectral mixture analysis (MESMA)
The purpose of the first paragraph in this section is unclear. Is it used to clean the ground data? How does it link to the process in R.
Please specify the QGIS plugin or function used

**Authors’ response**

Thank you for the comment. We have revised the sentence and clarified the use of Orfeo ToolBox (OTB) in QGIS accordingly, as reflected in lines 172–178.

**Comment 19:**

Section: Accuracy Assessment

The methodologies should be clearly stated. It is unclear which points were used for validation. While the text states that only ground points were used, based on earlier paragraph it seems a mix of ground points and points from high-resolution imagery interpretation were used. It also appears the validation sample could be taken in the same polygon as the calibration samples. This high correlation usually led to overestimation of the different accuracy metrics.

**Authors’ response**

Thank you for the insightful comment. We have clarified in the manuscript that only 228 independent GPS points were used for the MESMA classification accuracy assessment, as explained in the accuracy assessment section (lines 387–389).

**Comment 20:**

The current validation seems to be based on sample count. I strongly recommend doing a “map” validation. Where the user and producer accuracy are representative on the map content. Additionally, the validation method should allow the result to come with their corresponding confidence interval. One recommended method is described here:P. Olofsson and al.: Good practices for estimating area and assessing accuracy of land change.

**Authors’ response**

Thank you for the suggestion. We applied a probability-based sampling design with 95% confidence intervals calculated using exact binomial methods. Area estimates were derived from reference proportions to ensure robust and transparent validation. Revisions are reflected in lines 400–406 (Accuracy section), 485–489 (Results), and the updated Table 5.

**Comment 21:**

The validation sample size for coffee seems to be 0.3*340=102. But the count for each coffee type is not specified and potentially small (less than 30).

**Authors’ response**

Thank you for the comment. We initially used 340 pixel samples for overall Robusta coffee cropland classification, then reclassified into three Robusta coffee cropping systems using MESMA. An independent set of 228 points covering all classes was used solely for validation, revised in lines 387–389, Accuracy section.

**Comment 22:**

The author should discuss the fact the coffee has a lower producer accuracy than the other cover. It should be discussed if some specific coffee system is not well represented on the map. As this would impact the quality of the final product combining the 2 maps.

**Authors’ response**

Thank you for this important observation. We have noted in the manuscript that Robusta coffee cropland showed lower accuracy (PA = 59.4), likely due to spectral similarity with surrounding vegetation. This limitation and its implications are discussed in lines 513–518, as also noted by Reviewer #1.
**Comment 23:**

Line 240: The definition is not clear as the difference between class and categories is not explained.

**Authors’ response**

We thank the reviewer for this comment. For consistency and clarity, we have used the term “classes” throughout the entire manuscript to refer to the different land cover or crop types.

**Comment 23:**

Line 244: RMSE not explained. And OA seems to be defined only for the robusta analysis only.
**Authors’ response**

We have added a clear definition of RMSE in the accuracy assessment section, specifically in lines 396–398.

**Comment 24:**

Section: Results

Line 275: The sentence is difficult to follow and should be rephrased for clarity.

**Authors’ response**

We thank the reviewer for the suggestion. The sentence has been rephrased for clarity as recommended in line 438-446

**Comment 25:**
Section: Discussion
Line 329: The citation is repeated

**Authors’ response**

We thank the reviewer for pointing this out. The repeated citation on line 520 has been corrected.

**Comment 26:**

Line 325: As the composite is only based on median of one year of imagery, the statement that seasonal and interannual dynamic is captured should be justified.

**Authors’ response**

We thank the reviewer for the observation. The sentence has been revised to accurately reflect that only seasonal, not interannual, dynamics were captured in line 511-512

**Comment 27:**
Line 330-333: specify the country mapped in other studies

**Authors’ response**

Thank you for the suggestion. We have added the countries where the cited studies were conducted for clarity (line 522).

**Comment 28:**
Line 334: without confidence interval and with potentially different validation method the difference between the studies is probably not statistically significant.

**Authors’ response**

We thank the reviewer for the valuable comment. The manuscript has been revised in dicusstion lines 528-533

**Comment 29:**
Line 340-346: in table 3 UA=95% > PA=59.4% it seems either the texts or table have inverted UA and PA.

**Authors’ response**

Thank you for pointing this out. We acknowledge the error in the original text and have corrected it on lines 542, 543, and 545. The values presented in Table 4 are correct.

**Comment 30:**
Line 358: A low RMSE indicates good model fit but does not necessarily imply high classification accuracy.

**Authors’ response**

Thank you for the observation. The sentence has been revised accordingly on line 450.

**Comment 31:**

Line 362: While the two-step approach may intuitively improve accuracy, this was not empirically tested in the study.

**Authors’ response**

We appreciate the reviewer’s comment and have clarified in the text (lines 577–579) that the accuracy improvement from the two-step approach is expected, but it was not empirically tested in this study.

**Comment 32:**

It would be interesting to add a comment on the accuracy once the 2 steps are combined. As it seems the 2 tables are addressing one step at time.

**Authors’ response**

We appreciate this thoughtful observation. The two classification steps were presented separately: the first distinguishing major land use/land cover (LULC) types, and the second classifying Robusta coffee cropping systems (CS) within the Robusta coffee cropland identified in step one.

**Comment 33:**

Section: Conclusion

Line 400: The superiority of the proposed approach over a direct classification was not tested and should not be claimed without supporting evidence.

**Authors’ response**

We thank the reviewer for this valuable comment. We have removed the statement claiming superiority over direct classification, as no supporting evidence was provided in lines 642-644.

**Comment 34:**

The value of in-situ spectral data extraction versus extraction from Sentinel data was not explicitly assessed in the study. This point should be revised or supported with additional analysis.

**Authors’ response**

Thank you for your valuable comment. We agree that highlighting the comparative value of in-situ versus Sentinel-2 data is important. As suggested, we have now revised the Discussion section (lines 613–617) to address this point.

**Comment 35:**

Tables and Figures

Table 1: Consider adding example images for each cropping system.

**Authors’ response**

Thank you for the suggestion. We have added representative field photographs for the three Robusta coffee cropping systems in Fig. 4 to enhance clarity and visual interpretation

**Reviewer 2** – **Comment 36:**

The definition of "forest" should be refined. Terms like "normal natural forest" are vague. Specify whether this includes primary, secondary, or degraded forest.

**Authors’ response**

We thank the reviewer for pointing out this ambiguity. We have revised the terminology to clarify what is meant by “normal natural forest.” Specifically, we now define “natural forest” in our study in Table 2

**Comment 37:**

Clarify which categories contain non-coffee tree crops.

Overall, class definitions should be made more precise and operational.

**Authors’ response**

Thank you for this helpful comment. We have clarified which Robusta coffee cropping systems were considered in the Methods section of the revised manuscript (lines 301–311).

**Reviewer 2** – **Comment 38:**

Table 2:

Clarify what the numbers in the formula represent. Presumably, wavelength of the different bands?

**Authors’ response**

Thank you for your comment, and we have incorporated this clarification into the caption of Table 3 in the revised manuscript.

**Reviewer 2** – **Comment 39:**

Figure 5:

Use uniform scale for figures 1-3 and same figure size. Figure d) has a wrong horizontal axis.

**Authors’ response**

Thank you for the observation. We have revised Figure 7 to ensure a uniform scale and consistent figure size across panels a–f. Additionally, the horizontal axis in panel d) has been corrected to reflect the appropriate pixel fraction range.

**Reviewer 2** – **Comment 40:**

Figure 6:

The figure text is too small and hard to read. White on yellow contrast if hard to see. Use the same size for all the figures. And Some zoom would be interesting.

**Authors’ response**

Thank you for the feedback. We have increased the font size, improved color contrast, and standardized the formatting of all figures, including Fig. 7, to enhance readability.
