# Supplementary material for: Mapping Robusta coffee (Coffea canephora) cropping systems in Uganda: A two-step pixel and sub-pixel based approach with Sentinel-2 data
Source: PLoS One. 2026 Jan 5;21(1):e0338803. doi: 10.1371/journal.pone.0338803 (PMC12768349; doi:10.1371/journal.pone.0338803)
Supplement: Attachment — Submitted filename: Response to Reviewers_R.docx [file pone.0338803.s003.docx]

Mr. Getachew Kebede

Integrated Data and Analytics Platform (IDAP)

Tel.: +254208632280

Mob.: +254790224476

Email: [garagaw@icipe.org](mailto:garagaw@icipe.org)

Date: November 06, 2025

Dr. Emily Chenette,

Editor-in-Chief

Journal of PLOS One California, USA

**Subject: Submission of second revised paper (Manuscript Number: PONE-D-25-11912)**

Dear Dr. Emily Chenette,

We appreciate your time in handling our above-referenced manuscript entitled “Mapping Robusta coffee (*Coffea canephora*) cropping systems in Uganda: A two-step approach with Sentinel-2. We are also grateful for the valuable feedback from the editor and reviewers.

We have carefully revised the manuscript and addressed all comments from the editor and reviewers. In the revised version, changes are highlighted in yellow (Reviewer #3) and green (Reviewer #4). We hope that our revision and responses are adequate.

Sincerely,

Getachew Kebede Aragaw

Corresponding author

***Authors’ Point-by-Point Response to Reviewers’ Comments***

**Review#3 Comments to the Author**

**Reviewer #3**: The MS entitled ‘Mapping Robusta coffee (Coffea canephora) cropping systems in Uganda: A two-step pixel and sub-pixel based approach with Sentinel-2 data’ could be important because it advances methods to accurately map Uganda’s heterogeneous Robusta coffee systems, providing critical insights for climate adaptation, policy planning, and improving smallholder farmer livelihoods. The authors aimed to evaluate the use of MESMA with Sentinel-2 imagery to classify major LULC types, isolate Robusta coffee cropland, and map three distinct Robusta coffee cropping systems at a subpixel scale in Uganda. Although most sections of the manuscript are well-developed, certain revisions are necessary before it is ready for publication.

**Authors’ response**

We sincerely thank the reviewer for the positive and constructive feedback. We have carefully addressed all the comments, which significantly improved the quality of our manuscript.

**Reviewer #3 – Detailed point-by-point response**

**Comment 1:**

1. In line 22, the scientific name of Robusta coffee should be provided immediately after the term 'Robusta coffee,' rather than being repeated later in line 25.

**Authors’ response**

Addressed (lines 22 and 25), thank you for the suggestion.

**Comment 2:**

2. The sectional titles lack clear organization: some are included in figure captions, while others are associated with subtitles.

**Authors’ response**

Thank you for pointing this out. We have revised the manuscript to ensure consistent and clear organization of sectional titles, separating them from figure captions and aligning subtitles with the main text structure.

**Comment 3:**

3. In lines 100-101, ‘MESMA to the coffee class only to map the three CS at a subpixel scale’ is confusing to me. It would be nice if the authors would rewrite and clarify it.

**Authors’ response**

Thank you for the suggestion. We clarified the sentence in lines 106–110 to explain that MESMA was applied only within the Robusta coffee cropland to distinguish the three coffee cropping systems at a sub-pixel scale. The sentence now reads “We first classified the study landscape into five major LULC classes to distinguish Robusta coffee cropland from other land types. After isolating the coffee cropland, we applied MESMA exclusively within the coffee cropland class to further discriminate and map the three coffee CS at a subpixel scale”

**Comment 4:**

The socio-economic variables, like farmer practices, yield differences, or management intensity, could affect the results. The discussion would be more solid if the authors addressed these missing variables.

**Authors’ response**

Yes, that is right we agree with the reviewer and included this shortcoming in the Discussion section (lines 583–589) to read “Furthermore, socio-economic variables, such as farmer practices, crop management intensity, and yield differences, may could also contribute to unexplained spectral variability in the EMs. While such datasets were currently not available, integrating these information in future work could provide a more comprehensive understanding of Robusta coffee CS spectral heterogeneity for effective and accurate classification and mapping”.

**Comment 5:**

The study relies on a static image composite from a single dry season, making it unlikely to capture seasonal vegetation dynamics. The authors should consider revising these discussions or adding additional data to assess seasonal changes in vegetation.

**Authors’ response**

Thank you for this constructive criticism, which we agreed with. The same concern was also raised by reviewer #4. We discussed this as one of the study limitations in lines 594–596 “Seasonal variations in vegetation phenology, pest damage, and other biotic or abiotic stressors can significantly alter the spectral characteristics of both the LULC classes and the three coffee CS. These changes reduce their spectral separability and hinder accurate mapping when relying on a single image or spectral sampling from only one season. Therefore, future studies should incorporate multi-seasonal imagery and Ems derived from both dry and wet seasons, as well as from varying stress conditions, to enhance the robustness and reliability of classification accuracy”.

**Comment 6:**

Although the authors mention Robusta coffee productivity in the abstract and introduction, the study presents no results addressing it. The authors should either remove this content or provide additional data and analyses to demonstrate the study’s relevance to Robusta coffee productivity.

**Authors’ response**

Thank you for this observation. We agree and removed productivity from the abstract and introduction.

**Reviewer #4: Overall Assessment**

This manuscript presents an innovative two-step remote sensing approach for mapping Robusta coffee cropping systems (CS) in Uganda, integrating Random Forest classification of LULC with subpixel MESMA spectral unmixing. The combination of Sentinel-2 imagery with in-situ hyperspectral endmembers is novel for East African coffee systems and provides valuable applied insights for coffee management and climate adaptation.
The work is generally well designed and clearly presented. However, several methodological and interpretive issues require clarification, particularly regarding endmember representativeness, seasonal bias in spectral data, and the absence of comparisons with alternative classifiers or auxiliary predictors (e.g., climatic or topographic variables).

Addressing these points will considerably strengthen the manuscript.
Overall, this is a promising and publishable study, provided the authors undertake major revisions.

**Authors’ response**

We thank the reviewer for the encouraging and insightful comments. We appreciate the recognition of our study’s novelty and methodological contribution to mapping Robusta coffee systems in Uganda. In response, we have addressed all methodological and interpretive concerns, including clarifying endmember representativeness, accounting for potential seasonal bias, and discussing comparisons with alternative classifiers and auxiliary predictors. These revisions have substantially improved the quality of the manuscript.

**Strengths**

• **High relevance**: Uganda is a key Robusta producer, and mapping coffee systems has direct implications for adaptation to climate change and value-chain management (Bunn et al., 2019) https://cgspace.cgiar.org/bitstreams/bd1b904e-271c-4e50-ad35-55321bc02f5/download?utm_source=chatgpt.com]; Bukomeko et al., 2019) [https://doi.org/10.1007/s10457-017-0172-8].

• **Novel methodology**: The two-step design—Random Forest for LULC followed by MESMA at subpixel scale—demonstrates a creative approach rarely applied to coffee agroforestry mapping.
• **Integration of field hyperspectral data**: Using in-situ spectra to derive endmembers increases ecological realism and reduces reliance on generalized spectral libraries (Somers et al., 2011)[https://doi.org/10.1016/j.rse.2011.03.003].
• **Strong classification results**: The reported accuracies (93.5% LULC; 89.9% coffee CS) are notable given the highly heterogeneous smallholder landscape.

• **Transparency and data access**: The provision of open data resources supports reproducibility and potential reuse by other researchers.

**Reviewer #4 – Detailed point-by-point response**

**Major Concerns**

**Comment 1:**

• Endmember representativeness

o The analysis is based on three endmembers sampled during a single dry season. This may not fully capture the spectral variability introduced by phenology, pest damage, or stress conditions (Mutanga & Skidmore, 2004) [https://doi.org/10.1080/01431160310001654923].

o The authors should discuss how seasonal changes might bias classification and outline future improvements (e.g., inclusion of wet-season spectra).

**Authors’ response**

We thank the reviewer for this constructive criticism, which was alo raised by the first reviewer. We agree with both reviewers and included this shortcoming in the discussion chapter (lines 594–6005) “Seasonal variations in vegetation phenology, pest damage, and other biotic or abiotic stressors can significantly alter the spectral characteristics of both the LULC classes and the three coffee CS (73). These changes reduce their spectral separability and hinder accurate mapping when relying on a single image or spectral sampling from only one season. Therefore, future studies should incorporate multi-seasonal imagery and Ems derived from both dry and wet seasons, as well as from varying stress conditions, to enhance the robustness and reliability of classification accuracy”. The relevant references have also been added to the References section (lines 871–872).

**Comment 2:**

• Comparison with alternative methods

o MESMA is well established, but no comparison is made with alternative classifiers or subpixel approaches (e.g., SMA, SVM, or deep learning; Kussul et al., 2017) [https://doi.org/10.1109/LGRS.2017.2681128].

o Even if not implemented, the rationale for selecting MESMA over newer approaches should be expanded.

**Authors’ response**

We thank the reviewer, particularly for this comment and for referring us to such an interesting paper by Kussul et al., (66). The rationale of selecting MESMA is expanded as suggested by the reviewer (Furthermore, unlike the spectral mixture analysis, which uses a fixed set of endmembers for all pixels, MESMA allows EM combinations to vary for each pixel, making it more adaptable to spatially heterogeneous surfaces. While deep learning methods can also capture complex spectral–spatial relationships, they typically require large datasets and high computational resources. MESMA provides a more transparent, data-efficient, and interpretable approach for mapping mixed land-cover types, especially in areas with limited training data or where physical understanding of spectral behavior is essential.), please see lines 95-101 in the introduction. We also added this argument “Although alternative subpixel classification approaches, such as support vector machine (SVM) (74) and deep learning (75), could have also been applied in this study, but a direct comparison between MESMA and these methods in mapping coffee CS was beyond this study scope. Future studies should explore the performance of different subpixel classifiers in mapping coffee CSs in the discussion (lines 525-532)

”.

**Comment 3:**

• Validation strategy

o The validation relies on polygons from the same period and region. This may overestimate accuracy (Tariq et al., 2023) [https://doi.org/10.1080/10095020.2022.2100287]. Consideration of independent temporal or spatial datasets would strengthen reliability.

**Authors’ response**

We thank the reviewer for this comment. We agreed and provided some discussion sentences. “Similarly, validation polygon samples were obtained from the same period and region to assess the performance of our classification approach. This approach, while practical, may lead to a slight overestimation of classification accuracy (59). Future studies should employ independent validation datasets collected across different time periods and geographic locations to enhance the reliability and robustness of the classification assessment.” in the discussion (lines 600-606)

**Comment 4:**

•Contextualization within broader agroecological debates

o The discussion could connect more explicitly with recent literature showing how heterogeneity in cropping systems influences remote sensing detectability and ecosystem outcomes (e.g., Escobar-López et al., 2022 [https://doi.org/10.3390/rs14163847]; Hunt et al., 2020) [https://doi.org/10.3390/rs12122041].

**Authors’ response**

We thank the reviewer for this comment. We addressed this in lines 546–551 “However, our study area represents a highly heterogeneous cropping system, where the structural and compositional diversity of Robusta coffee CSs influences both classification performance and ecological outcomes. This relationship aligns with broader agroecological evidence showing that variations in cropping system heterogeneity strongly affect remote sensing detectability as well as landscape-scale ecological functions (5, 13)”.

**Comment 5:**

o In particular, Cassamo et al. (2022)[https://doi.org/10.1016/j.agee.2022.108341] emphasize that mapping cropping systems provides a foundation for assessing ecosystem services and sustainability in smallholder agroecosystems. This perspective complements the current work and underscores its potential applications beyond classification, for example in policy design and environmental assessments.

**Authors’ response**

We thank the reviewer for this valuable suggestion. In lines 491–499, we have expanded the discussion to emphasize the broader relevance of our findings, noting, as highlighted by Cassamo et al. (2022), that mapping cropping systems provides a foundation for assessing ecosystem services, sustainability, and policy applications “Importantly, mapping Robusta coffee CSs extends beyond simple land-cover classification. As noted by Cassamo et al. (70), detailed characterization of cropping systems forms the foundation for assessing ecosystem services and sustainability within smallholder agroecosystems. This highlights the broader policy and environmental relevance of our findings for coffee-producing regions, where spatially explicit insights can inform sustainable land management and climate-resilient agricultural planning.”

**Minor Concerns**

**Comment 6:**

• Methodological detail: Some processing steps (e.g., spectral resampling in hsdar, MESMA implementation in RStoolbox) could be described more explicitly for reproducibility.

**Authors’ response**

We have revised the processing stems of spectral resampling and MESMA for explicit description (lines 285-292).

**Comment 7:**

• Figures: Several figures (Figs. 5–7) would benefit from clearer legends and larger font size.

**Authors’ response**

We have addressed the comment by improving the clarity of the figures. Specifically, Fig. 7 has been split into two separate graphs for better readability: Fig. 9a now presents the frequency of pixel fraction values and threshold sensitivity, while Fig. 9b shows the spectral angle mapper (SAM) values, quantifying spectral separability. Additionally, we have increased the font size of axis titles, labels, and legends across all figures to enhance clarity.

**Comment 8:**

• Terminology consistency: Ensure consistent use of “coffee cropping systems (CS)” rather than alternating with “coffee systems.”

**Authors’ response**

Addressed, thank you.

**Comment 9:**

• References: Update with recent contributions on coffee/agroforestry mapping (Escobar-López et al., 2022; Kelley et al., 2018 [https://doi.org/10.3390/rs10060952]).

**Authors’ response**

We have cited all these references, thank you for referring us to such interesting papers, which we learned a lot from them.

**Comment 10:**

• Language polishing: Minor English corrections needed (e.g., “the Robusta CS class was masked” → “the Robusta coffee class was masked”).

**Authors’ response**

Thank you for pointing this out. However, “the Robusta coffee cropland class was masked” is the correct phrasing (see lines 28–29), and we have ensured consistency throughout the manuscript. We also polished the manuscript for other language error.

**Comment 11:**

The manuscript makes a strong methodological and applied contribution, but requires clarification of uncertainties (seasonality, validation limits), expanded discussion of alternatives, and broader contextualization with recent agroecological literature.
Importantly, the recommendation of “major changes” reflects the need for expanded analysis and framing, not a flaw in the current methodology. With revisions, the study can significantly advance understanding of coffee cropping system mapping in Africa

**Authors’ response**

We appreciate the positive assessment of our methodology and contribution. We have addressed the concerns by clarifying uncertainties (seasonality, validation limits), expanding the discussion of alternative approaches, with recent agroecological literature to strengthen and advance the overall framing understanding of coffee cropping system mapping in Africa.
